# Warming but Not Nitrogen Addition Alters the Linear Relationship Between Microbial Respiration and Biomass

**DOI:** 10.3389/fmicb.2019.01055

**Published:** 2019-05-10

**Authors:** Hui Wei, Xiaomei Chen, Jinhong He, Letong Huang, Weijun Shen

**Affiliations:** ^1^ Key Laboratory of Agro-Environment in the Tropics, Ministry of Agriculture, South China Agricultural University, Guangzhou, China; ^2^ School of Geographical Sciences, Guangzhou University, Guangzhou, China; ^3^ Key Laboratory of Vegetation Restoration and Management of Degraded Ecosystems, South China Botanical Garden, Chinese Academy of Sciences, Guangzhou, China; ^4^ University of Chinese Academy of Sciences, Beijing, China

**Keywords:** heterotrophic soil respiration, soil organic matter decomposition, warming, atmospheric N deposition, subtropical forest

## Abstract

Soil contains a large amount of organic matter, which constitutes the largest terrestrial carbon pool. Heterotrophic or microbial respiration (R_h_) that results from microbial decomposition of soil organic carbon (SOC) constitutes a substantial proportion of soil C efflux. Whether soil microbial biomass is of primary importance in controlling R_h_ remains under debate, and the question of whether the microbial biomass-decomposition relationship changes with warming and nitrogen (N) deposition has rarely been assessed. We conducted an incubation experiment to test the relationship between R_h_ and the size of soil microbial communities in two layers of soil collected from a natural subtropical forest and to examine whether the relationship was affected by changes in temperature and by added N in different forms. The results showed that regardless of the added N species, the N load did not significantly affect R_h_ or the size of the soil microbial communities. These results could be due to a long-term N-rich soil condition that acclimates soil microbial communities to resist N inputs into the studied forest; however, warming may significantly stimulate SOC decomposition, reducing soil microbial biomass under high temperatures. A significant linear soil microbial biomass-decomposition relationship was observed in our study, with the coefficients of determination ranging from 54 to 70%. Temperature rather than N additions significantly modified the linear relationship between soil microbial biomass and respiration. These results suggest that warming could impose a more substantial impact than N addition on the relationship between soil microbial biomass and SOC decomposition.

## Introduction

In the terrestrial biosphere, soil reserves the greatest amount of carbon (C), with top soil to a depth of 1 m contributing approximately two-thirds (approximately 1,500 Pg) of the global C stock (Batjes, 1996; Scharlemann et al., 2014; Lal, 2018). The size of the soil C pool is even larger than that of the combined biotic and atmospheric C pools. Stabilizing such a large C pool is critical to balancing the global C cycle and stabilizing the global climate system (Friedlingstein et al., 2010; Melillo et al., 2017); therefore, many attempts have been made to study topics related to soil organic carbon (SOC), e.g., quantifying the global and regional C stocks, exploring the key influential factors, and predicting SOC dynamics under global changes.

Soil, as the largest terrestrial C pool, emits approximately 100 Pg CO_2_-C into the atmosphere per year (Bond-Lamberty and Thomson, 2010; Jian et al., 2018), primarily through the microbial SOC decomposition that constitutes ~63% of global soil respiration (Bond-Lamberty et al., 2018). The decomposition of SOC, which has often been used interchangeably with heterotrophic respiration in the literature (Kirschbaum, 2006), greatly influences the stock of soil C and its feedback to climate (Stewart et al., 2007; Bond-Lamberty et al., 2018). Temperature and moisture are of primary importance for determining the rate of SOC decomposition in most ecosystems, since they directly affect the size and activity of SOC decomposers. Moreover, temperature and moisture can regulate multiple processes, such as the absorption of enzymes and SOC on the soil surface, indirectly affecting SOC decomposition (Davidson and Janssens, 2006; Conant et al., 2011; Zhou et al., 2014). Soil texture (especially clay content) and substrate availability are frequently advanced as controlling factors of SOC decomposition and have therefore received considerable attention (Wang et al., 2003; von Lützow et al., 2006; Birge et al., 2015). As direct decomposition agents, soil microbial communities are recognized as likely playing critical roles in SOC decomposition by adjusting the community size, composition, and function in changing habitats (Ramirez et al., 2012; Whitaker et al., 2014; Huang et al., 2018).

However, the relationship between decomposition and the soil microbial community size (hereafter abbreviated as the decomposition-biomass relationship) remains inconclusive, with limited and contradictory observations (Stockmann et al., 2013; Saleem, 2015). For instance, Ramirez et al. (2012) observed that SOC decomposition was altered less than soil microbial biomass (on average −11 vs. −35%) over a 1-year-long soil incubation with N additions and Liu and Greaver (2010) found a similar pattern from a global perspective (−8 vs. −20%). These results suggest that N deposition could change the decomposition-biomass relationship. Moreover, a recent study showed that SOC decomposition and the total amount of microbial phospholipid fatty acids (PLFAs) responded differently to the added N load (Chen et al., 2019), implying that changing N deposition could affect the decomposition-biomass relationship. While the size of soil microbial communities has been regarded as a main predictor of the SOC decomposition rate (Colman and Schimel, 2013; Garcia-Palacios et al., 2015; Ali et al., 2018) and explicitly presenting its dynamics may improve the predictive performance of soil C models (Hararuk et al., 2015), some authors argue that its inclusion in biogeochemical models is less important (Wang et al., 2003; Birge et al., 2015). Obviously, this variation in the decomposition-biomass relationship impedes a consistent conclusion.

Global warming and atmospheric N deposition are among the primary issues of global change (Galloway et al., 2008; Gutknecht et al., 2012; IPCC, 2013), and their ecological consequences still need to be addressed. In spite of the debate over potential mechanisms (e.g., changes in substrate supply vs. microbial acclimation), it is almost a consensus that SOC decomposition can be stimulated by warming temperatures over a short period of time and then return to its previous level after a certain duration of warming. In many cases, soil microbial biomass has been observed to decrease under warming conditions relative to control treatments. These observations imply that warming can modify the decomposition-biomass relationship, but empirical evidence is still needed. Previous studies demonstrate that atmospheric N deposition can positively, negatively, or not affect SOC decomposition in different ecosystems, with the responses controlled by other factors (such as soil substrate supply and forest productivity) (Janssens et al., 2010; Eberwein et al., 2015; Chen et al., 2019). Compared with the increasing atmospheric N load, changes in the deposited N species have received little attention, although they have been widely observed (Liu et al., 2013; Hunova et al., 2017). Furthermore, warming and N deposition may interactively affect SOC decomposition (Eberwein et al., 2017; Fang et al., 2018), and the response of SOC decomposition to N deposition likely depends on the input N species (Wang et al., 2018). However, whether the microbial biomass-decomposition relationship would change with warming and N deposition has rarely been assessed.

In the present study, temperature and N species were considered as two treatment factors, and their main and interactive effects on SOC decomposition and the potential decomposition-biomass relationship were observed. Soil samples were collected from surface and subsurface layers in an old-growth evergreen broadleaved forest and incubated. In nature, the upper soil layers are firstly affected by atmospheric environmental changes compared to deep soils. Although experimentally incubating soils in the lab presents potential limitations, two soils that had different properties were considered to be two contrasting treatments and were used to observe the soil microbial response to determine the effects of the initial soil properties. We expected a linear relationship to exist between the soil microbial decomposition and community size in both soils considering that soil microorganisms are the final decomposers of SOC. Temperature and N species were included as two treatment factors, mainly to clarify whether these important environmental changes altered the decomposition-biomass relationship in soils.

## Materials and Methods

### Experimental Design

The studied soils were collected at two soil depths (0–10 and 10–20 cm) from a mature forest located in the Dinghushan Nature Reserve (112°30′39″–112°33′41″E, 23°09′21″–23°11′30″N) in southern China. The study site has a tropical monsoon climate zone with typical hot rainy and cold dry seasons. The mean annual air temperature is 22.3°C, and average annual precipitation is approximately 1,600 mm, of which nearly 80% occurs in the hot rainy season from April to September and the remainder in the cold dry season. The studied forest is a climax forest that has naturally developed over 400 years with few anthropogenic disturbances. Evergreen broadleaved tree species, such as *Castanopsis chinensis*, *Schima superba*, and *Cryptocarya chinensis*, are the dominant species. The soil is categorized as an oxisol according to the USDA soil taxonomy (Tang et al., 2006). The surface and subsurface soils that were collected for use in this study had significantly different soil physiochemical and microbial properties, with the surface soil possessing a lower pH but a substantially higher soil microbial biomass and nutrient concentrations than the subsurface soil (Wei et al., 2017). The two layers of soil were considered to be two soil types of contrasting soil properties for the purpose of determining the treatment effects of warming and N addition across soils, which would indicate whether the treatment effects change as soil properties changed.

This incubation experiment had a three-factor full factorial design, with soil type, incubation temperature, and N form as the three independent variables. In particular, two soils (the surface and sub-surface soils), three incubation temperatures (10, 20, and 30°C), and four forms of N (control with no N addition, nitrate N, ammonium N, and urea N) were employed, producing a total of 24 combinations of experimental treatments. For each treatment, four composite soils collected in each of four 15 m × 15 m quadrats were treated as four replicates in the incubation and statistical analyses. The incubation temperatures fell within the seasonal temperature range at the study site (3.2–36.5°C; Wei et al., 2015). N addition was applied at 3.79 mg N 50 g^−1^ dry soil, approximately equivalent to that of the regional N deposition load (50 kg N ha^−1^ y^−1^; Fang et al., 2008). Further information regarding the experimental design can be found in a recent parallel study by Wei et al. (2017).

### Incubation Experiment and Sample Analyses

After collection, the composited soil samples were immediately transferred to the laboratory and mixed by passing through a 2 mm soil sieve. Visible plant residue and rocks were removed in this pretreatment process. Based on the aforementioned experimental design (2 soils × 3 incubation temperatures × 4 N forms × 4 replicates), a total of 96 fresh soil samples were prepared. Samples of 50 g oven-dried-base soils were placed into a 200 mL triangular glass flask to incubate for 90 days. Three thermostat incubators (RXZ-600B, Southeast Instrument Co., Ltd., Ningbo, China) were set up at the three incubation temperatures. The soil water content within the incubation period was maintained at 55% of the water holding capacity by adding deionized water after periodically weighing the containers to determine water loss. This level of soil moisture falls within the optimal moisture range for the soil microbial activities in the studied forest (Zhou et al., 2014). Soils were aerobically incubated with a ball of cotton plugging the flasks to minimize water loss while permitting gas exchange.

The soil CO_2_ efflux rate was measured 12 times over the 90-day incubation period. For each measurement, a rubber stopper was used to seal each flask and two headspace gas samples were collected with 30 min separating the two collections. The gas samples were immediately analyzed to determine the CO_2_ concentration on an Agilent 7890A gas chromatograph with a flame ionization detector (Agilent Technologies Inc., Palo Alto, California, USA), and increases in the CO_2_ concentration over time were used to estimate the soil CO_2_ efflux rate.

At the end of incubation, the soil microbial biomass of each sample was determined using two methods, the chloroform fumigation method and the PLFAs method. In brief, the chloroform fumigation and extraction method were conducted as proposed by Vance et al. (1987), and the C concentration in the extracts was determined on a Total Organic Carbon Analyzer (TOC-VCSH, Shimadzu Corp., Kyoto, Japan) with an extraction efficiency coefficient of 0.45 (Wu et al., 1990). PLFAs were isolated from soil samples as described by Bossio and Scow (1998), with a mixture of chloroform, methanol, and citrate as the buffering solution. Phospholipids were collected using a silica column (500 mg, ANPEL Laboratory Technologies Inc., Shanghai, China), and then, the total amount of PLFAs in each sample was determined using an Agilent 7890 gas chromatograph equipped with a Sherlock Microbial Identification system (version 6.2, MIDI Inc., Newark, Delaware, USA) after methanolysis by adding a mildly alkaline solution. An internal standard fatty acid (19:0) was mixed into each sample to quantify the content of each PLFA, and the sum of the contents of all the PLFAs was determined as the total amount of PLFAs (nmol g^−1^) for each sample.

### Data Analyses

An integrating method was used to calculate the amount of CO_2_ emissions during the experimental period (Wetterstedt et al., 2009). Tests of normality were conducted using the Shapiro-Wilk method. Original data were used for further analyses if the normality assumption was met; otherwise, data were rank transformed to obtain normal scores for subsequent analyses. Three-way analyses of variance (ANOVA) were conducted to detect the main and interactive effects of soil, incubation temperature, and N addition on soil CO_2_ emission or microbial biomass. One-way ANOVAs were employed to test the significant effects of N treatments. Due to the nonsignificant N effects observed in most of the treatment combinations (Tables 1 and 2), we did not categorize samples based on the N treatments for subsequent analyses. The effects of the incubation temperature were tested by one-way ANOVAs, while those of the soil type were detected using an independent-sample *T* test. Linear regression was conducted to verify the relationships between the two methods of soil microbial biomass or between soil CO_2_ emission and microbial biomass. Partial correlation analysis was used to double check the significance of the relationship between SOC decomposition and microbial biomass, with the SOC content serving as a controlling factor. The significance level was set at *p* ≤ 0.05. All analyses were conducted using IBM SPSS Statistics 22 (IBM Corp., New York, USA), and graphs were made with SigmaPlot 10.0 (Systat Software Inc., California, USA).

**Table 1:** Statistical *F* and significance level *p* derived from three-way analyses of variances with the incubation temperature (T), nitrogen addition (N), and soil (S) as the three independent factors.

	CO_2_ emission		MBC		PLFAs		CO_2_/PLFAs	
	*F*	*p*	*F*	*p*	*F*	*p*	*F*	*p*
T	163.97	<0.001	20.58	<0.001	10.11	<0.001	106.32	<0.001
N	2.29	0.085	1.91	0.135	0.31	0.818	1.01	0.394
S	144.69	<0.001	124.32	<0.001	156.71	<0.001	2.89	0.094
T × N	1.11	0.367	2.69	0.021	0.15	0.988	0.58	0.743
T × S	3.51	0.035	5.58	0.006	1.35	0.265	6.86	0.002
N × S	0.79	0.506	1.34	0.270	0.06	0.982	0.12	0.946
T × N × S	2.20	0.053	1.61	0.158	0.10	0.996	0.093	0.997

*MBC, microbial biomass carbon; PLFAs, the total amount of phospholipid fatty acids; CO_2_/PLFAs, the CO_2_ emissions standardized by PLFAs (μg nmol^−1^ PLFAs)*.

**Table 2:** Statistical *F* and significance level *p* derived from one-way analyses of variances with nitrogen addition as the independent factor.

Temp. (°C)	Soil layer (cm)	CO_2_ emission		MBC		PLFAs		CO_2_/PLFAs	
		*F*	*p*	*F*	*p*	*F*	*p*	*F*	*p*
10	0–10	2.89	0.080	1.61	0.238	0.018	0.996	10.27	0.001
	10–20	1.64	0.233	4.03	0.034	0.039	0.989	0.79	0.524
20	0–10	0.25	0.857	1.08	0.396	0.57	0.645	2.32	0.127
	10–20	0.91	0.466	5.12	0.017	0.054	0.982	0.40	0.756
30	0–10	0.17	0.916	1.45	0.277	0.12	0.949	0.47	0.710
	10–20	0.12	0.945	2.50	0.109	0.28	0.842	0.26	0.854

*MBC, microbial biomass carbon; PLFAs, the total amount of phospholipid fatty acids; CO_2_/PLFAs, CO_2_ emissions standardized by PLFAs (μg nmol^−1^ PLFAs)*.

## Results

Three-way ANOVAs showed that the incubation temperature and soil type had significant effects on the accumulated CO_2_ emissions, MBC, and total amount of PLFAs (*p* < 0.001 for all), while N addition, regardless of the applied forms, did not significantly affect the soil CO_2_ emissions and microbial biomass as indicated by MBC and PLFAs (*p* > 0.05; Table 1). The interactive effects between two of the three factors (i.e., temperature, N form, and soil type) had mostly nonsignificant effects on the accumulated CO_2_ emissions, MBC, or PLFAs, with exception of the interaction of temperature and soil type on CO_2_ emissions (*p* = 0.035) and that of temperature and N addition or soil type on soil MBC (*p* = 0.021 and 0.006, respectively; Table 1). To further verify the nonsignificant N effects, one-way ANOVAs were conducted to compare the group means of the accumulated soil CO_2_ emissions and microbial biomass at each of the three temperatures in one of the two soils (Table 2). The results showed that the N additions did not significantly alter the accumulated soil CO_2_ emissions and microbial biomass in most cases (*p* > 0.05), except for MBC in the subsurface soil at 10 and 20°C (*p* = 0.034 and 0.017, respectively; Table 2). We further standardized the CO_2_ emissions by the amount of soil microbial PLFAs to indicate the relative microbial activity per unit of soil microbial biomass. The resultant index of CO_2_/PLFAs showed a similar pattern with the total CO_2_ emissions (Tables 1 and 2).

**Figure 1:**
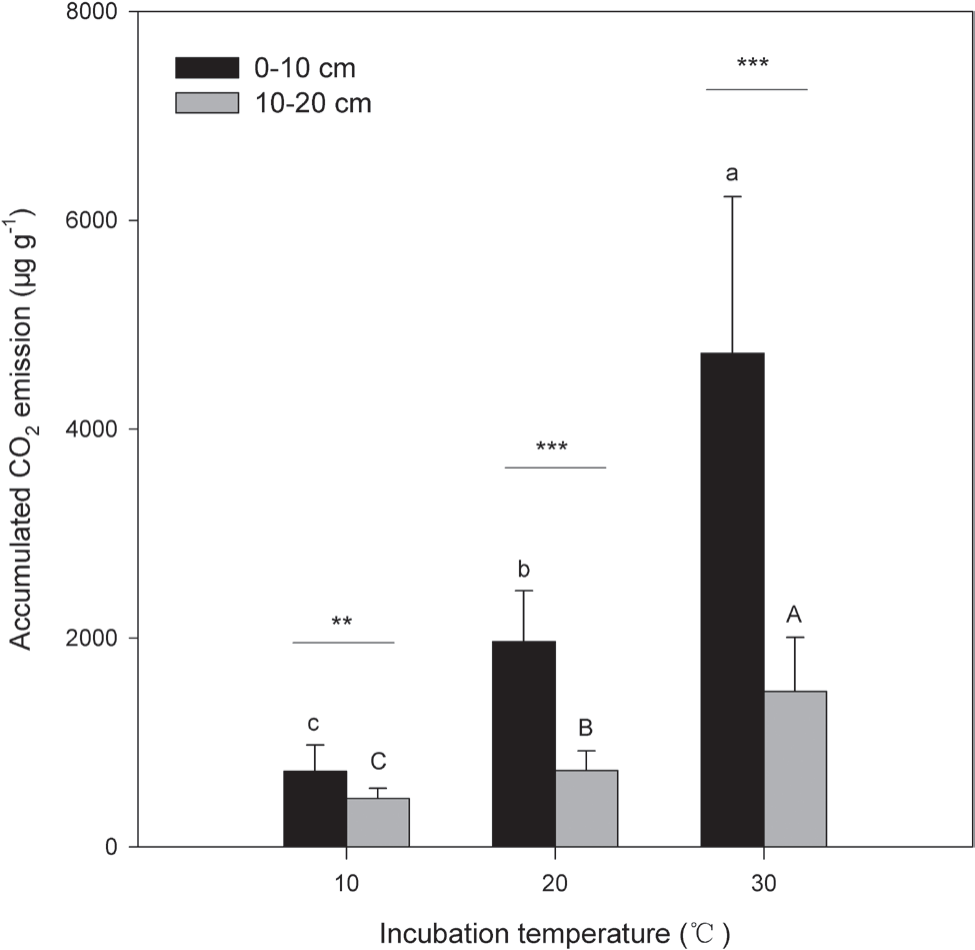
Accumulated amount of CO_2_ emissions from surface (0–10 cm) and subsurface (10–20 cm) soils within the investigation period. The bars are the average values, and the error bars are the standard deviations (*n* = 4). Different lowercase letters above the bars indicate significant differences among the temperature treatments for surface soil, while uppercase letters indicate significance for subsurface soil. Double and triple stars indicate significant differences between soils under each of the incubation temperatures at significance levels of *p* < 0.01 or *p* < 0.001, respectively.

**Figure 2:**
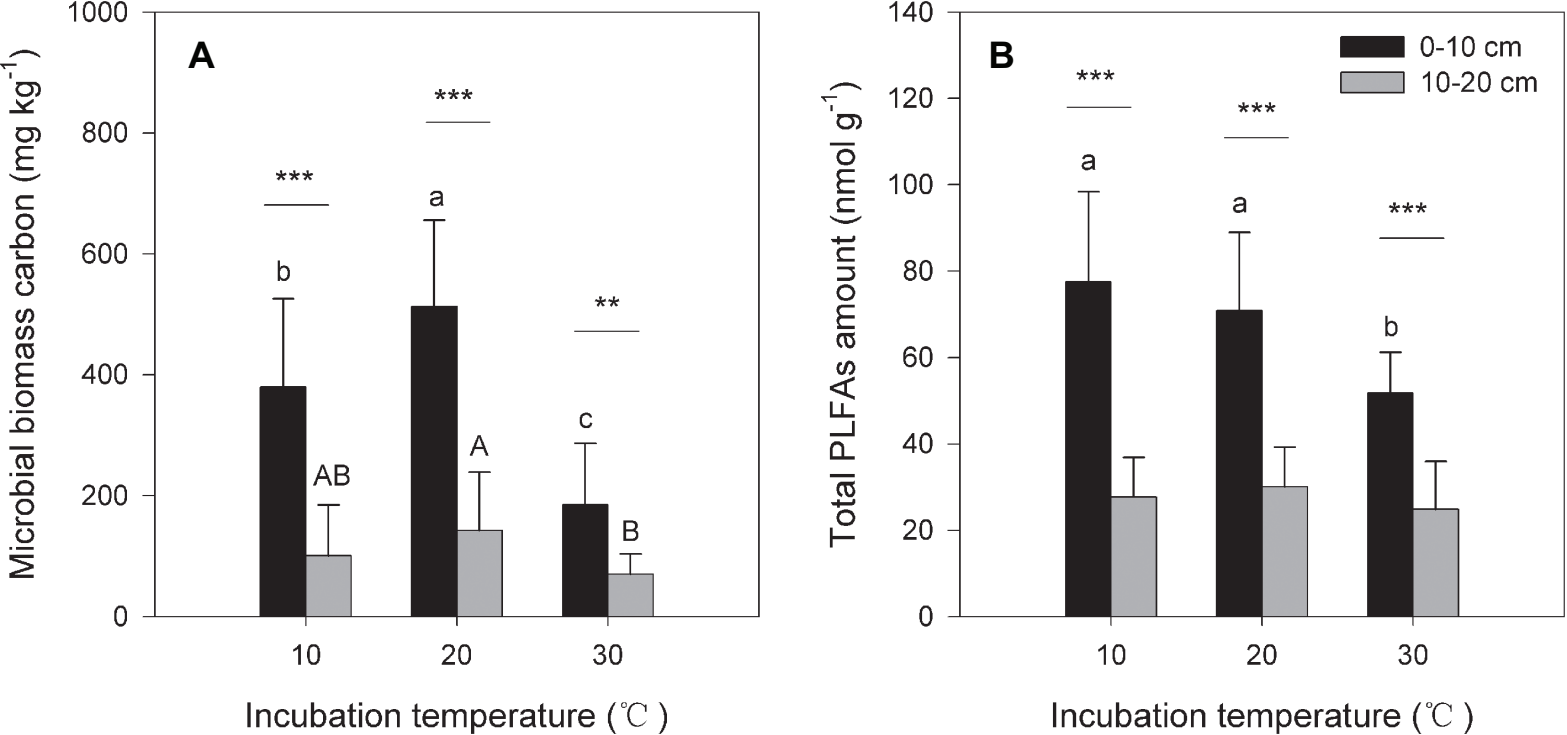
Soil microbial biomass carbon [MBC **(A)**] and total amount of phospholipid fatty acids [PLFAs **(B)**] under different incubation temperatures. The bars are the average values, and the error bars are the standard deviations (*n* = 4). Different lowercase letters above the bars indicate significant differences among the temperature treatments for surface soil, while uppercase letters indicate significance for subsurface soil. Double and triple stars indicate significant differences between the soils under each of the incubation temperatures at significance levels of *p* < 0.01 or *p* < 0.001, respectively.

**Figure 3:**
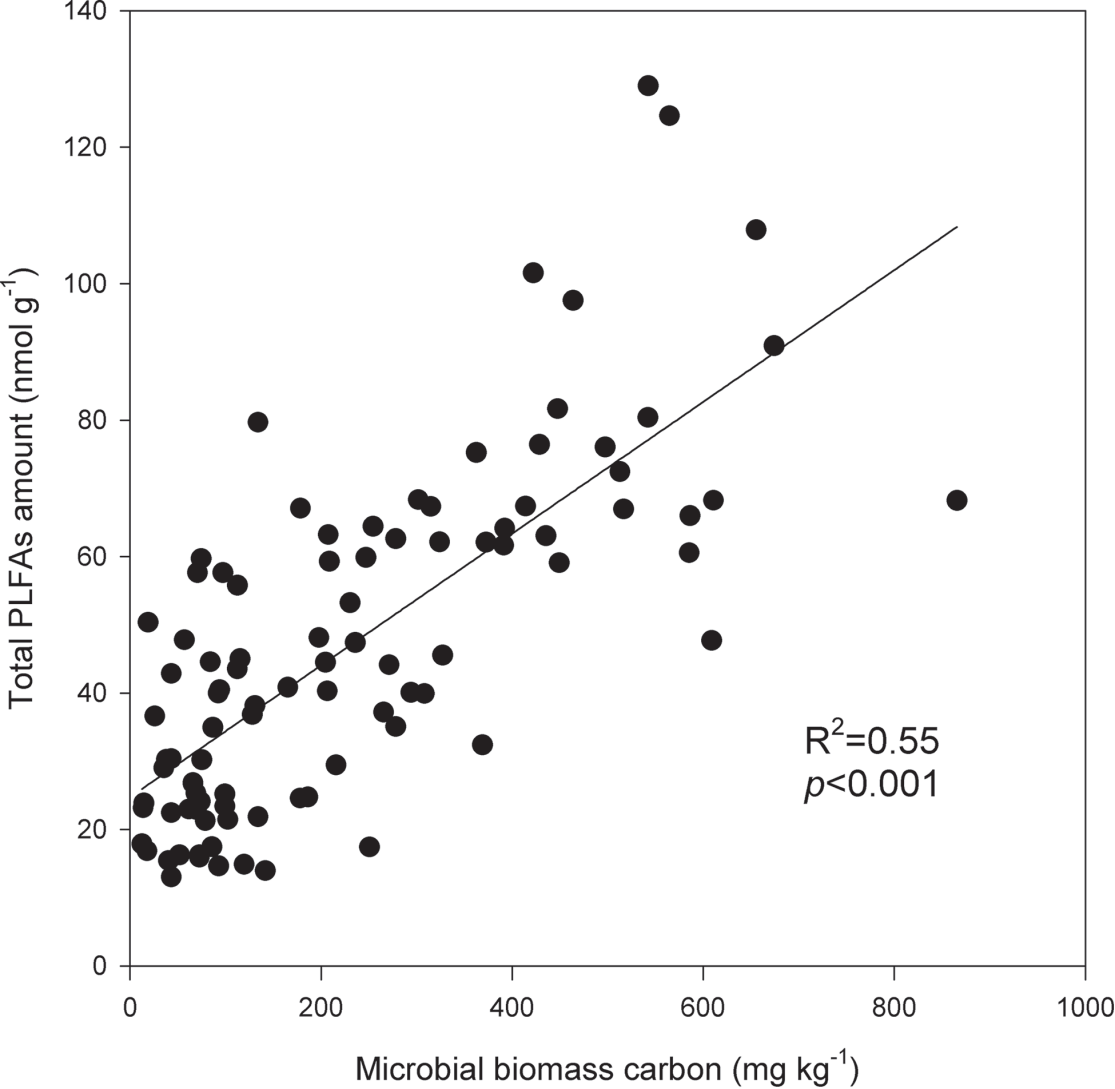
Linear relationship between the total amounts of phospholipid fatty acids (PLFAs) and soil microbial biomass carbon. The plots are observation values, and the solid line is the linear regression line. The coefficient of determination *R*^2^ and significance level *p* are presented.

Under the three incubation temperatures, accumulated CO_2_ emissions were consistently higher from the surface soil than from the subsurface soil within the incubation period, and the surface soil CO_2_ emissions responded at a greater magnitude to the increasing incubation temperature (*p* = 0.035; Table 1; Figure 1). There were significant exponential increases in accumulated soil CO_2_ emissions in both soils as the incubation temperature increased (*p* < 0.05; Figure 1). Likewise, we observed significant differences in soil microbial biomass between the surface and subsurface soils, which were indicated by the consistent pattern of the soil MBC content and total amount of PLFAs between the two soils (*p* < 0.05; Figure 2). Contrary to the soil CO_2_ emissions, soil microbial biomass was significantly lower at the higher incubation temperature (i.e., 30°C) than at 10 or 20°C in the two soils (*p* < 0.05), except for a case in the subsurface soil that contained a comparable amount of total PLFAs at all three temperatures (*p* > 0.05; Figure 2B). Moreover, the soil MBC content had a significant linear relationship with the total amount of PLFAs (*p* < 0.001; Figure 3), mutually verifying the reliability of the two methods to determine soil microbial biomass.

**Figure 4:**
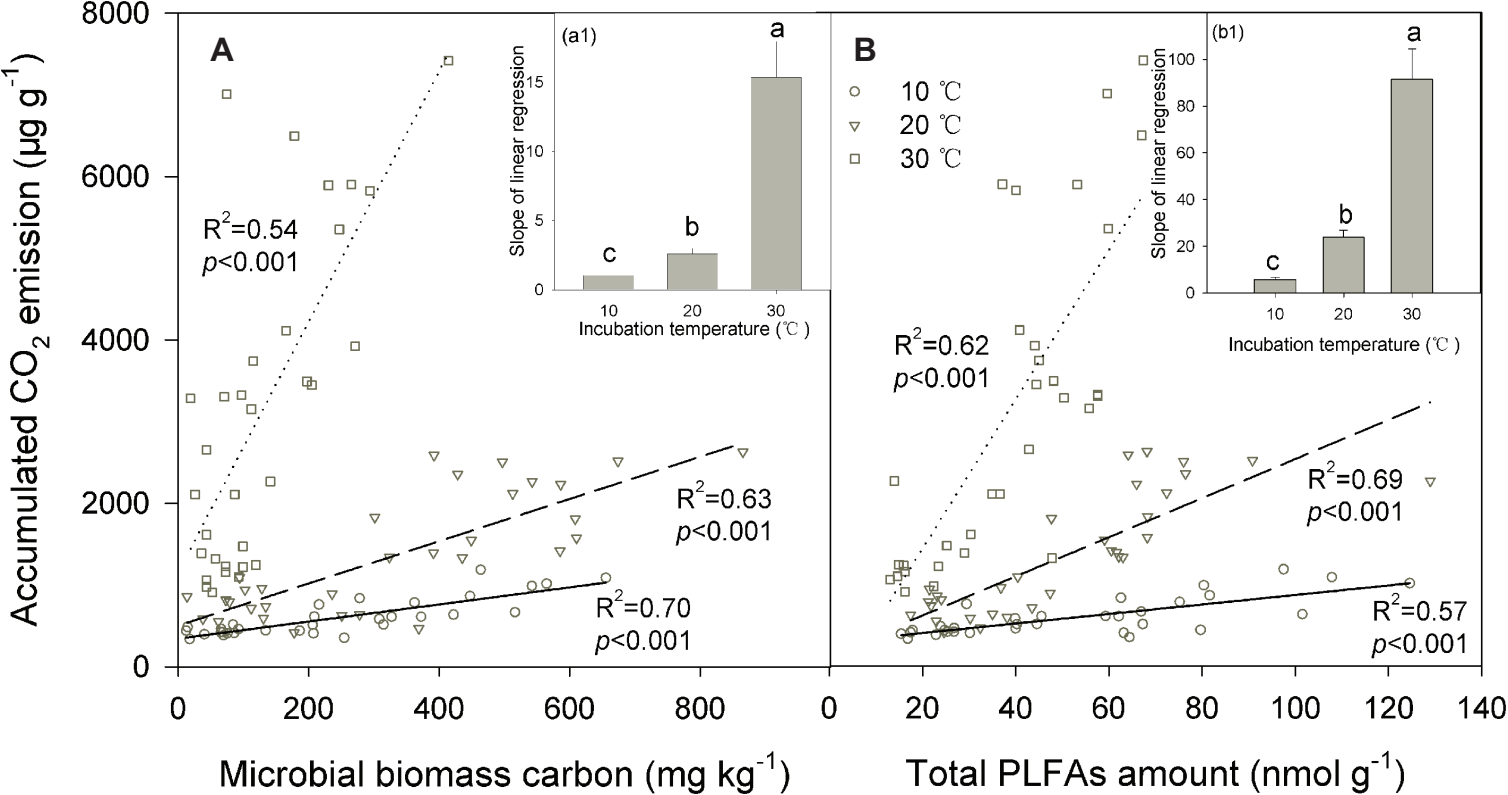
Linear relationship between the accumulated amount of soil CO_2_ emissions and microbial biomass carbon **(A)** and the total amount of phospholipid fatty acids [PLFAs **(B)**] under different incubation temperatures, with the inserted panels A1 and B1 presenting the slopes of linear regressions under the three incubation temperatures. In the figure, the solid lines are regression lines between the two variables observed at 10°C, while the dashed and potted lines are those at 20 and 30°C, respectively. The coefficient of determination *R*^2^ and significance level *p* are presented for each incubation temperature. Different lowercase letters above the bars in the inserted panels A1 and B1 indicate significant differences among the temperatures.

We conducted linear regressions to detect bivariate correlations between soil CO_2_ emissions and microbial biomass across soils. The results showed that soil microbial biomass was significantly positively correlated with the accumulated soil CO_2_ emissions (*p* < 0.001 for all cases; Figure 4). The coefficient of determination of these linear regressions ranged between 0.54 and 0.70 for the MBC content and between 0.57 and 0.69 for PLFAs, suggesting that changes in soil microbial biomass may explain most of the total variation in soil CO_2_ emissions across soils. This pattern was further verified by the significant correlations between SOC decomposition and MBC or total PLFAs in the partial correlation analyses, where the SOC content served as a control to exclude the interactive effect of the SOC content (indicating substrate supply) on its decomposition (*p* < 0.05 for all). Incubation temperature significantly modified the slope of these linear regressions between the accumulated soil CO_2_ emissions and microbial biomass (*p* < 0.001; Figures 4A,B), and the slope index exponentially increased as the incubation temperature increased (*p* < 0.001; Figures 4A1,B1). This pattern was consistent for the two indicators of soil microbial biomass, MBC, and PLFAs (Figures 4A,B).

## Discussion

In the present study, N additions did not significantly alter the size and activity of soil microbial communities (Tables 1 and 2). This result may be attributable to the N-rich soil conditions of the study site and long-term high N deposition (Fang et al., 2008; Chen et al., 2015) that causes soil microbial communities to acclimate to N-rich soil conditions. We observed that the soil microbial PLFAs composition was not significantly altered by these types of N addition in our parallel study (Wei et al., 2017), suggesting microbial resistance to current N deposition in N-rich forest soils; however, increasing atmospheric N deposition (Liu et al., 2013) could result in significant changes in soil microbial communities because soil microorganisms could nonlinearly respond to N addition, with a N load threshold above which N addition will significantly affect soil microbial activity and size (Zhou et al., 2012). This threshold, however, may be context specific. For example, Ramirez et al. (2010) found that soil microbes in a variety of temperate mountainous ecosystems were resistant to N addition at different levels and that this microbial resistance more or less depended on the added N species. Therefore, N species may work only when the N load exceeds the threshold of microbial N resistance in the studied ecosystems. By contrast, we found that the total CO_2_ emissions were significantly stimulated by an increased temperature (Table 1; Figure 1). This increase, however, may not be due to changes in the soil microbial community size because soil microbial biomass was significantly reduced under a high temperature of 30°C (Figure 2). This increase is more likely attributable to temperature-induced changes in the microbial community composition (Wei et al., 2017) and microbial physiology, as indicated by the standardized CO_2_ emissions by dividing the total amount of PLFAs (Table 1), i.e., the CO_2_ emissions per unit of soil microbial biomass.

Soil CO_2_ released during the investigation period was significantly higher in surface soil than in subsurface soil (Table 1; Figure 1), which was accompanied by a consistent pattern of the biomass of soil microbial communities across the soils (Figure 2). Consequently, this pattern produces a significant decomposition-biomass relationship (Figure 4) as expected, suggesting microbial control of SOC decomposition is mainly due to adjusting the biomass of soil microbial communities. Although soil microbial communities were significantly different in surface soil than those in subsurface soil, as presented in our parallel study (Wei et al., 2017), our observations of significant linear regressions with high coefficients of determination (all *R*^2^ > 0.50) support the hypothesis that the biomass of soil microbial communities may be of primary importance to control SOC decomposition (Fang and Moncrieff, 2005; Colman and Schimel, 2013; Ali et al., 2018), at least in this type of N-rich forest soil. This hypothesis is supported by the significant soil effect on the total CO_2_ emissions but insignificant effect on the CO_2_/PLFAs index (Table 1). This scenario could be attributed to the close correlations between the soil microbial community size and both the enzyme kinetic parameters (e.g., Michaelis constant *K*_m_) and its catalytic efficiency along with soil depth (Parvin et al., 2018), because microbial activity very likely depends on the enzymatic properties (Conant et al., 2011).

Such a decomposition-biomass relationship should be variable depending on environmental conditions. As observed in the present study, temperature may significantly modify the decomposition-biomass relationship, with significantly higher C emission potential per unit of soil microbial biomass at high temperatures (Figure 4). The correspondence of the slope index to the metabolic quotient reflects a cell eco-physiological entity and may rapidly respond to environmental stresses (Wardle and Ghani, 1995; Anderson, 2003; Saleem, 2015). Increasing the temperature could result in higher C and energy investments to maintain the stability of microbial cells and then in a higher metabolic quotient as a result of metabolic activation (Alvarez et al., 1995). The changing metabolic activity of soil microbial communities mainly contributed to the increasing trend of the slope index with temperature increases (Figure 4), probably as a result of temperature stimulation of microbial physiology, activity, and C utilization efficiency (Schimel et al., 2007; Saleem, 2015), which is associated with the temperature sensitivity of key parameters involved in microbial C processes (Davidson and Janssens, 2006; Wieder et al., 2013). Moreover, soil microbial activity and function may be related to the soil microbial community composition (Wei et al., 2014; Chen et al., 2019), and thus, shifts in the soil microbial community composition among temperature treatments (Wei et al., 2017) may induce modifications in the relationship between the size and function of the soil microbial community.

By contrast, the size of soil microbial communities does not account for the increase in the slope index with temperature (Figure 4) because soil microbial biomass is significantly lower at higher (e.g., 30°C) compared with lower temperatures (Figure 2). The same pattern of reduced soil microbial biomass under warming temperatures has been frequently observed in both field and incubation experiments (Lipson et al., 2000; Frey et al., 2008; Wei et al., 2014). This scenario could be attributable to altered substrate availability and microbial C assimilation efficiency or self-consumption of soil microbial biomass at high temperatures (Alvarez et al., 1995; Frey et al., 2008; Conant et al., 2011). Finally, warming induced increments in soil CO_2_ emissions are more likely due to increases in microbial physiological activity, which is supported by the increased CO_2_/PLFAs index indicating the activity per unit of soil microorganisms. This change in soil microbial physiological efficiency could be related to the shifts in soil microbial community composition revealed by the PLFAs analysis in our parallel study (Wei et al., 2017).

## Conclusions

The current N load did not significantly affect SOC decomposition, regardless of the changed N species, whereas increased temperature significantly stimulated soil microbial activity. The SOC decomposition rate showed a good correlation with the soil microbial community size, as determined by both the fumigation and PLFAs methods. This result suggests that soil microbial communities control SOC decomposition mainly by adjusting the size of microbial communities. However, warming temperatures could significantly affect the decomposition-biomass relationship. Combined with the observed decline in microbial biomass at high temperatures, our results indicate that warming-induced increases in microbial CO_2_ production are most likely due to altered microbial physiological activity. These results indicate a high dependence of SOC decomposition on the size of the soil microbial community, but this dependence is vulnerable to environmental changes, such as global warming.

## Author Contributions

WS and HW conceived the experiment. HW, XC, and JH conducted the study. HW, XC, and LH analyzed the data and wrote the draft manuscript. All authors contributed to result discussion and manuscript revision.

### Conflict of Interest Statement

The authors declare that the research was conducted in the absence of any commercial or financial relationships that could be construed as a potential conflict of interest.
